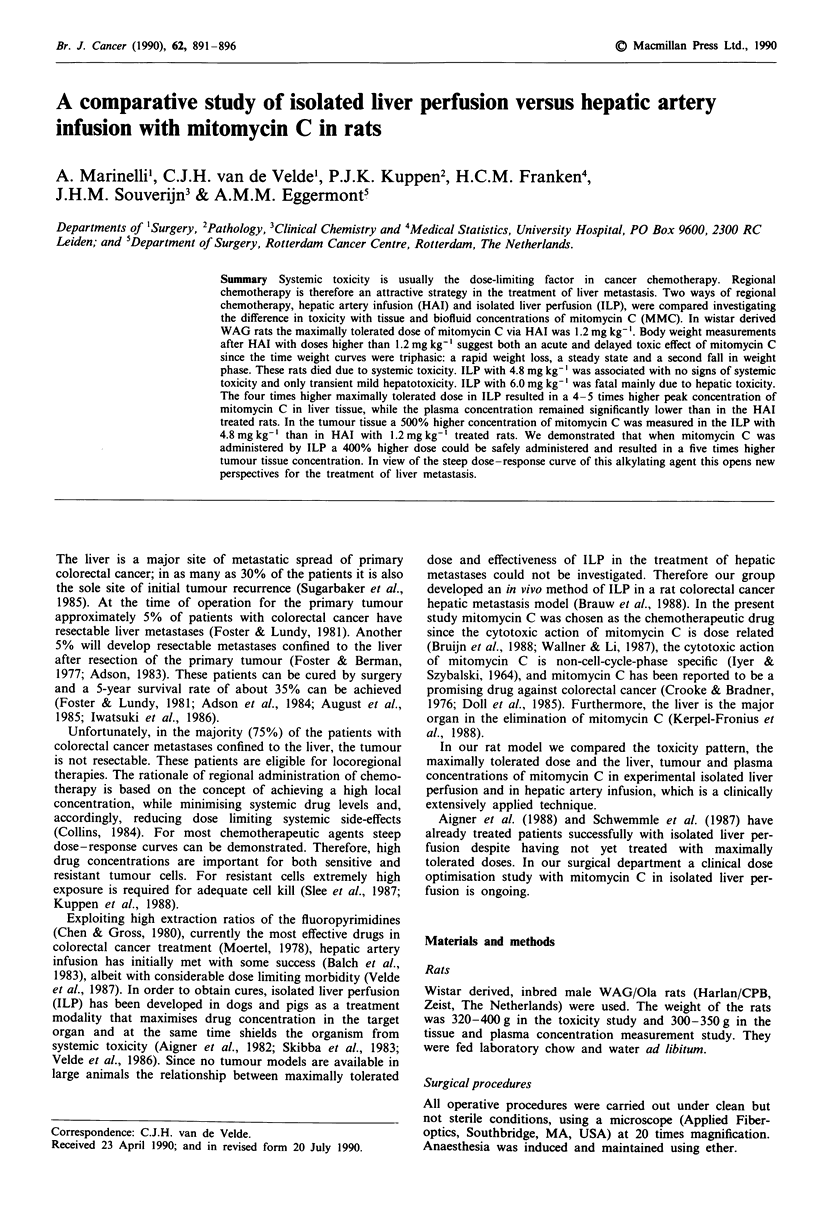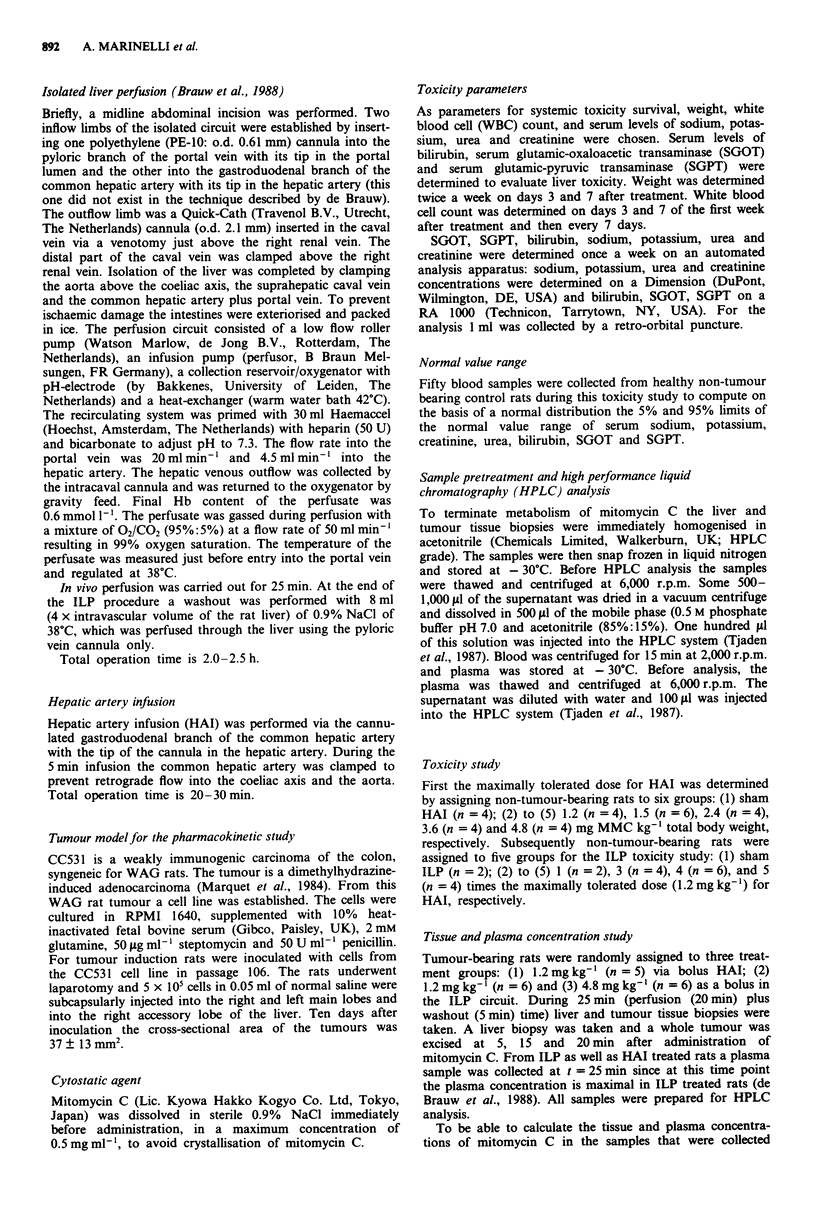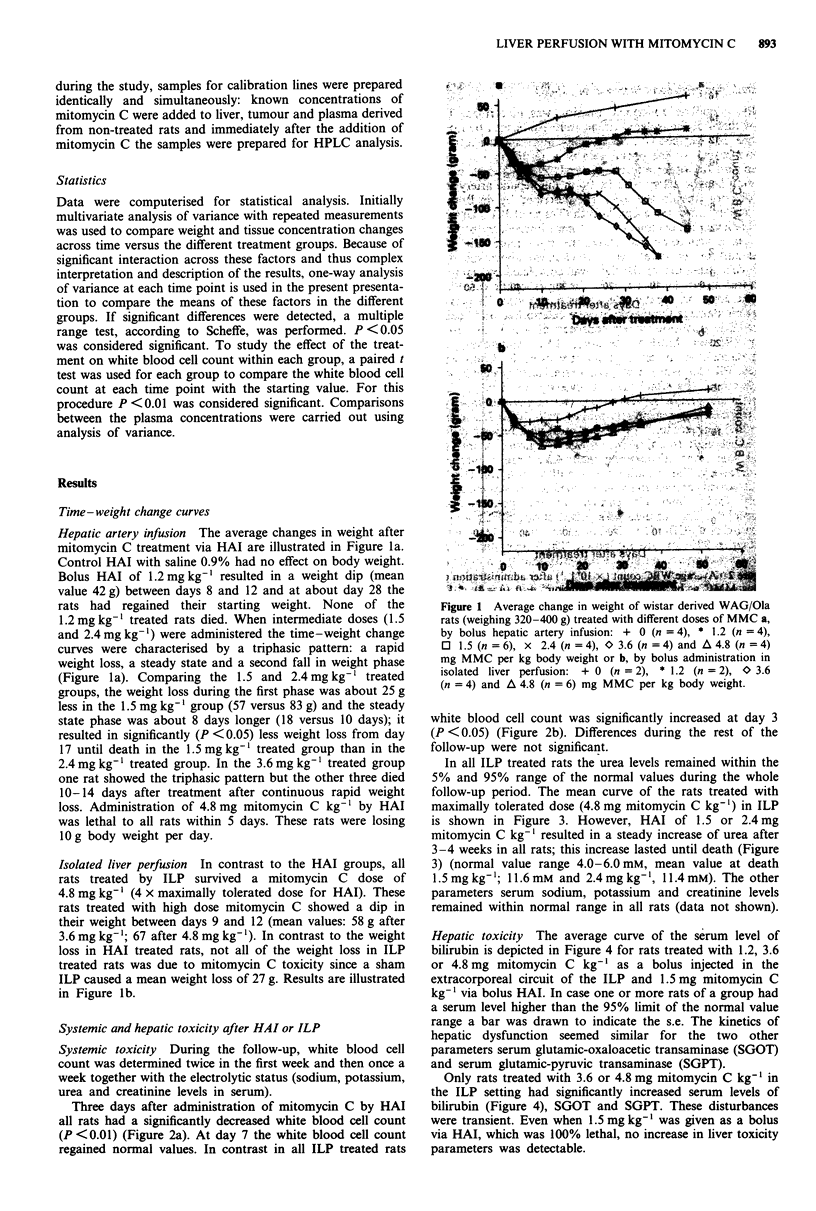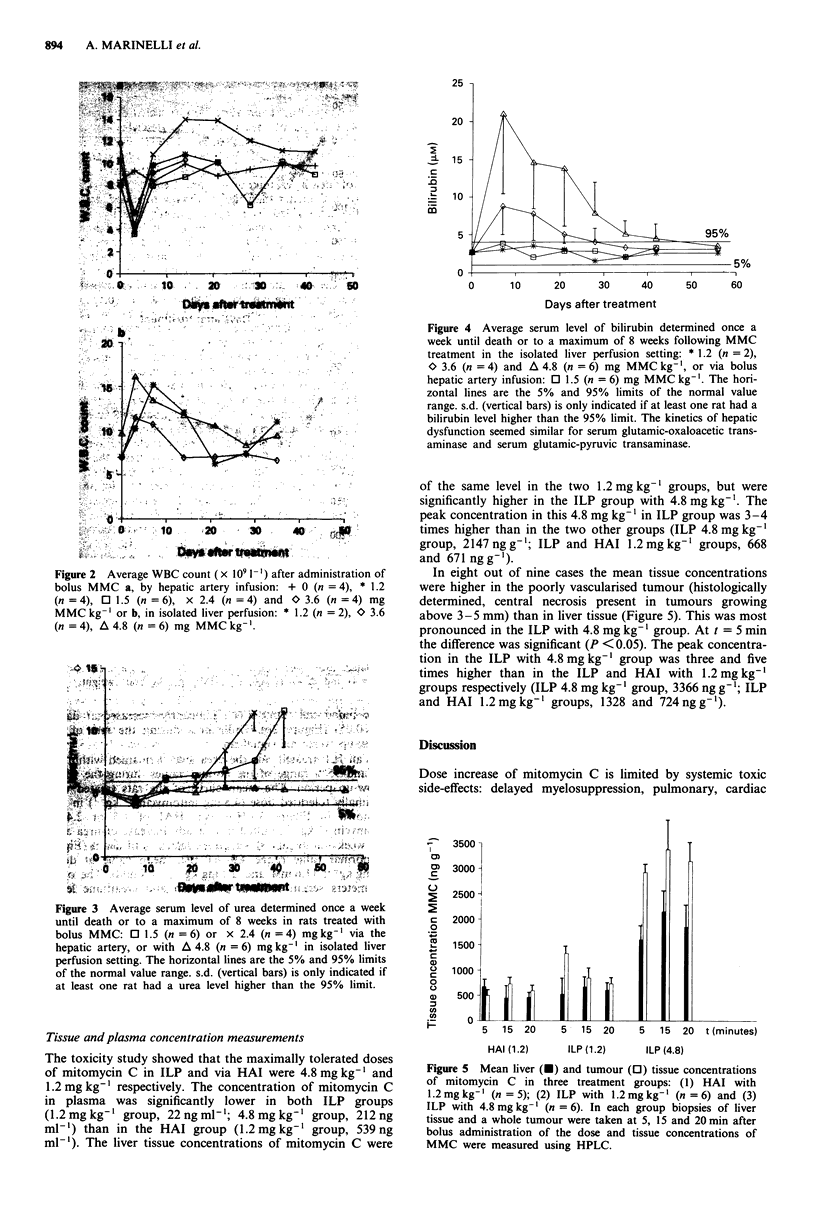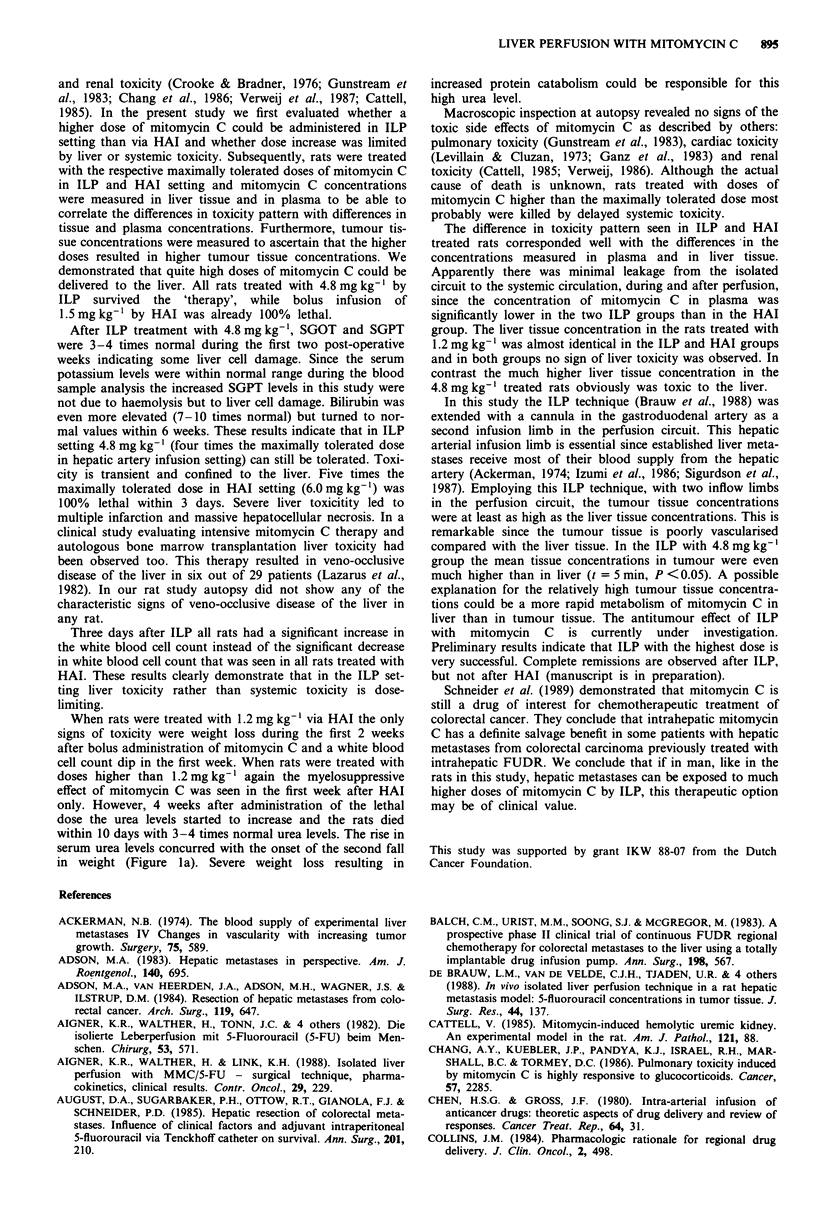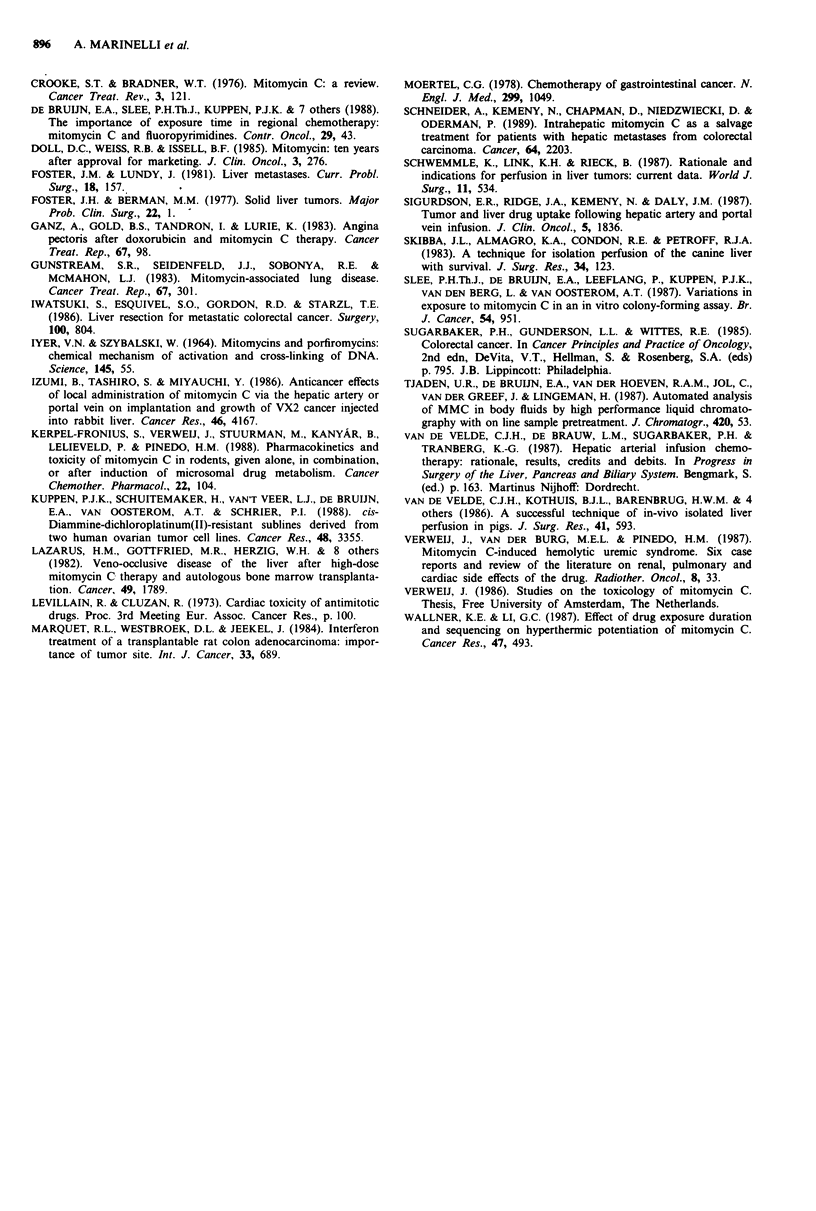# A comparative study of isolated liver perfusion versus hepatic artery infusion with mitomycin C in rats.

**DOI:** 10.1038/bjc.1990.404

**Published:** 1990-12

**Authors:** A. Marinelli, C. J. van de Velde, P. J. Kuppen, H. C. Franken, J. H. Souverijn, A. M. Eggermont

**Affiliations:** Department of Surgery, University Hospital, Leiden, The Netherlands.

## Abstract

Systemic toxicity is usually the dose-limiting factor in cancer chemotherapy. Regional chemotherapy is therefore an attractive strategy in the treatment of liver metastasis. Two ways of regional chemotherapy, hepatic artery infusion (HAI) and isolated liver perfusion (ILP), were compared investigating the difference in toxicity with tissue and biofluid concentrations of mitomycin C (MMC). In wistar derived WAG rats the maximally tolerated dose of mitomycin C via HAI was 1.2 mg kg-1. Body weight measurements after HAI with doses higher than 1.2 mg kg-1 suggest both an acute and delayed toxic effect of mitomycin C since the time weight curves were triphasic: a rapid weight loss, a steady state and a second fall in weight phase. These rats died due to systemic toxicity. ILP with 4.8 mg kg-1 was associated with no signs of systemic toxicity and only transient mild hepatotoxicity. ILP with 6.0 mg kg-1 was fatal mainly due to hepatic toxicity. The four times higher maximally tolerated dose in ILP resulted in a 4-5 times higher peak concentration of mitomycin C in liver tissue, while the plasma concentration remained significantly lower than in the HAI treated rats. In the tumour tissue a 500% higher concentration of mitomycin C was measured in the ILP with 4.8 mg kg-1 than in HAI with 1.2 mg kg-1 treated rats. We demonstrated that when mitomycin C was administered by ILP a 400% higher dose could be safely administered and resulted in a five times higher tumour tissue concentration. In view of the steep dose-response curve of this alkylating agent this opens new perspectives for the treatment of liver metastasis.


Br. J. Cancer (1990), 62, 891-896 © Macmillan Press Ltd., 1990

### A comparative study of isolated liver perfusion versus hepatic artery infusion with mitomycin C in rats

A. Marinelli[1], C.J.H. van de Velde[1], P.J.K. Kuppen[2], H.C.M. Franken[4], J.H.M. Souverijn[3] & A.M.M. Eggermont[5]

*Departments of [1]Surgery, [2]Pathology, [3]Clinical Chemistry and [4]Medical Statistics, University Hospital, PO Box 9600, 2300 RC Leiden; and [5]Department of Surgery, Rotterdam Cancer Centre, Rotterdam, The Netherlands.*

**Summary** Systemic toxicity is usually the dose-limiting factor in cancer chemotherapy. Regional chemotherapy is therefore an attractive strategy in the treatment of liver metastasis. Two ways of regional chemotherapy, hepatic artery infusion (HAI) and isolated liver perfusion (ILP), were compared investigating the difference in toxicity with tissue and biofluid concentrations of mitomycin C (MMC). In wistar derived WAG rats the maximally tolerated dose of mitomycin C via HAI was 1.2 mg kg$^{-1}$. Body weight measurements after HAI with doses higher than 1.2 mg kg$^{-1}$ suggest both an acute and delayed toxic effect of mitomycin C since the time weight curves were triphasic: a rapid weight loss, a steady state and a second fall in weight phase. These rats died due to systemic toxicity. ILP with 4.8 mg kg$^{-1}$ was associated with no signs of systemic toxicity and only transient mild hepatotoxicity. ILP with 6.0 mg kg$^{-1}$ was fatal mainly due to hepatic toxicity. The four times higher maximally tolerated dose in ILP resulted in a 4-5 times higher peak concentration of mitomycin C in liver tissue, while the plasma concentration remained significantly lower than in the HAI treated rats. In the tumour tissue a 500% higher concentration of mitomycin C was measured in the ILP with 4.8 mg kg$^{-1}$ than in HAI with 1.2 mg kg$^{-1}$ treated rats. We demonstrated that when mitomycin C was administered by ILP a 400% higher dose could be safely administered and resulted in a five times higher tumour tissue concentration. In view of the steep dose-response curve of this alkylating agent this opens new perspectives for the treatment of liver metastasis.

The liver is a major site of metastatic spread of primary colorectal cancer; in as many as 30% of the patients it is also the sole site of initial tumour recurrence (Sugarbaker *et al.*, 1985). At the time of operation for the primary tumour approximately 5% of patients with colorectal cancer have resectable liver metastases (Foster & Lundy, 1981). Another 5% will develop resectable metastases confined to the liver after resection of the primary tumour (Foster & Berman, 1977; Adson, 1983). These patients can be cured by surgery and a 5-year survival rate of about 35% can be achieved (Foster & Lundy, 1981; Adson *et al.*, 1984; August *et al.*, 1985; Iwatsuki *et al.*, 1986).

Unfortunately, in the majority (75%) of the patients with colorectal cancer metastases confined to the liver, the tumour is not resectable. These patients are eligible for locoregional therapies. The rationale of regional administration of chemotherapy is based on the concept of achieving a high local concentration, while minimising systemic drug levels and, accordingly, reducing dose limiting systemic side-effects (Collins, 1984). For most chemotherapeutic agents steep dose-response curves can be demonstrated. Therefore, high drug concentrations are important for both sensitive and resistant tumour cells. For resistant cells extremely high exposure is required for adequate cell kill (Slee *et al.*, 1987; Kuppen *et al.*, 1988).

Exploiting high extraction ratios of the fluoropyrimidines (Chen & Gross, 1980), currently the most effective drugs in colorectal cancer treatment (Moertel, 1978), hepatic artery infusion has initially met with some success (Balch *et al.*, 1983), albeit with considerable dose limiting morbidity (Velde *et al.*, 1987). In order to obtain cures, isolated liver perfusion (ILP) has been developed in dogs and pigs as a treatment modality that maximises drug concentration in the target organ and at the same time shields the organism from systemic toxicity (Aigner *et al.*, 1982; Skibba *et al.*, 1983; Velde *et al.*, 1986). Since no tumour models are available in large animals the relationship between maximally tolerated dose and effectiveness of ILP in the treatment of hepatic metastases could not be investigated. Therefore our group developed an *in vivo* method of ILP in a rat colorectal cancer hepatic metastasis model (Brauw *et al.*, 1988). In the present study mitomycin C was chosen as the chemotherapeutic drug since the cytotoxic action of mitomycin C is dose related (Bruijn *et al.*, 1988; Wallner & Li, 1987), the cytotoxic action of mitomycin C is non-cell-cycle-phase specific (Iyer & Szybalski, 1964), and mitomycin C has been reported to be a promising drug against colorectal cancer (Crooke & Bradner, 1976; Doll *et al.*, 1985). Furthermore, the liver is the major organ in the elimination of mitomycin C (Kerpel-Fronius *et al.*, 1988).

In our rat model we compared the toxicity pattern, the maximally tolerated dose and the liver, tumour and plasma concentrations of mitomycin C in experimental isolated liver perfusion and in hepatic artery infusion, which is a clinically extensively applied technique.

Aigner *et al.* (1988) and Schwemmle *et al.* (1987) have already treated patients successfully with isolated liver perfusion despite having not yet treated with maximally tolerated doses. In our surgical department a clinical dose optimisation study with mitomycin C in isolated liver perfusion is ongoing.

### Materials and methods

### *Rats*

Wistar derived, inbred male WAG/Ola rats (Harlan/CPB, Zeist, The Netherlands) were used. The weight of the rats was 320-400 g in the toxicity study and 300-350 g in the tissue and plasma concentration measurement study. They were fed laboratory chow and water *ad libitum*.

### *Surgical procedures*

All operative procedures were carried out under clean but not sterile conditions, using a microscope (Applied Fiberoptics, Southbridge, MA, USA) at 20 times magnification. Anaesthesia was induced and maintained using ether.

Correspondence: C.J.H. van de Velde.
Received 23 April 1990; and in revised form 20 July 1990.

*Isolated liver perfusion (Brauw et al., 1988)*

Briefly, a midline abdominal incision was performed. Two inflow limbs of the isolated circuit were established by inserting one polyethylene (PE-10: o.d. 0.61 mm) cannula into the pyloric branch of the portal vein with its tip in the portal lumen and the other into the gastroduodenal branch of the common hepatic artery with its tip in the hepatic artery (this one did not exist in the technique described by de Brauw). The outflow limb was a Quick-Cath (Travenol B.V., Utrecht, The Netherlands) cannula (o.d. 2.1 mm) inserted in the caval vein via a venotomy just above the right renal vein. The distal part of the caval vein was clamped above the right renal vein. Isolation of the liver was completed by clamping the aorta above the coeliac axis, the suprahepatic caval vein and the common hepatic artery plus portal vein. To prevent ischaemic damage the intestines were exteriorised and packed in ice. The perfusion circuit consisted of a low flow roller pump (Watson Marlow, de Jong B.V., Rotterdam, The Netherlands), an infusion pump (perfusor, B Braun Melsungen, FR Germany), a collection reservoir/oxygenator with pH-electrode (by Bakkenes, University of Leiden, The Netherlands) and a heat-exchanger (warm water bath 42°C). The recirculating system was primed with 30 ml Haemaccel (Hoechst, Amsterdam, The Netherlands) with heparin (50 U) and bicarbonate to adjust pH to 7.3. The flow rate into the portal vein was 20 ml $min^{-1}$ and 4.5 ml $min^{-1}$ into the hepatic artery. The hepatic venous outflow was collected by the intracaval cannula and was returned to the oxygenator by gravity feed. Final Hb content of the perfusate was 0.6 mmol $l^{-1}$. The perfusate was gassed during perfusion with a mixture of $O_2/CO_2$ (95%:5%) at a flow rate of 50 ml $min^{-1}$ resulting in 99% oxygen saturation. The temperature of the perfusate was measured just before entry into the portal vein and regulated at 38°C.

*In vivo* perfusion was carried out for 25 min. At the end of the ILP procedure a washout was performed with 8 ml (4 × intravascular volume of the rat liver) of 0.9% NaCl of 38°C, which was perfused through the liver using the pyloric vein cannula only.

Total operation time is 2.0–2.5 h.

*Hepatic artery infusion*

Hepatic artery infusion (HAI) was performed via the cannulated gastroduodenal branch of the common hepatic artery with the tip of the cannula in the hepatic artery. During the 5 min infusion the common hepatic artery was clamped to prevent retrograde flow into the coeliac axis and the aorta. Total operation time is 20–30 min.

*Tumour model for the pharmacokinetic study*

CC531 is a weakly immunogenic carcinoma of the colon, syngeneic for WAG rats. The tumour is a dimethylhydrazine-induced adenocarcinoma (Marquet *et al.*, 1984). From this WAG rat tumour a cell line was established. The cells were cultured in RPMI 1640, supplemented with 10% heat-inactivated fetal bovine serum (Gibco, Paisley, UK), 2 mM glutamine, 50 μg $ml^{-1}$ steptomycin and 50 U $ml^{-1}$ penicillin. For tumour induction rats were inoculated with cells from the CC531 cell line in passage 106. The rats underwent laparotomy and $5 \times 10^5$ cells in 0.05 ml of normal saline were subcapsularly injected into the right and left main lobes and into the right accessory lobe of the liver. Ten days after inoculation the cross-sectional area of the tumours was $37 \pm 13$ $mm^2$.

*Cytostatic agent*

Mitomycin C (Lic. Kyowa Hakko Kogyo Co. Ltd, Tokyo, Japan) was dissolved in sterile 0.9% NaCl immediately before administration, in a maximum concentration of 0.5 mg $ml^{-1}$, to avoid crystallisation of mitomycin C.

*Toxicity parameters*

As parameters for systemic toxicity survival, weight, white blood cell (WBC) count, and serum levels of sodium, potassium, urea and creatinine were chosen. Serum levels of bilirubin, serum glutamic-oxaloacetic transaminase (SGOT) and serum glutamic-pyruvic transaminase (SGPT) were determined to evaluate liver toxicity. Weight was determined twice a week on days 3 and 7 after treatment. White blood cell count was determined on days 3 and 7 of the first week after treatment and then every 7 days.

SGOT, SGPT, bilirubin, sodium, potassium, urea and creatinine were determined once a week on an automated analysis apparatus: sodium, potassium, urea and creatinine concentrations were determined on a Dimension (DuPont, Wilmington, DE, USA) and bilirubin, SGOT, SGPT on a RA 1000 (Technicon, Tarrytown, NY, USA). For the analysis 1 ml was collected by a retro-orbital puncture.

*Normal value range*

Fifty blood samples were collected from healthy non-tumour bearing control rats during this toxicity study to compute on the basis of a normal distribution the 5% and 95% limits of the normal value range of serum sodium, potassium, creatinine, urea, bilirubin, SGOT and SGPT.

*Sample pretreatment and high performance liquid chromatography (HPLC) analysis*

To terminate metabolism of mitomycin C the liver and tumour tissue biopsies were immediately homogenised in acetonitrile (Chemicals Limited, Walkerburn, UK; HPLC grade). The samples were then snap frozen in liquid nitrogen and stored at − 30°C. Before HPLC analysis the samples were thawed and centrifuged at 6,000 r.p.m. Some 500–1,000 μl of the supernatant was dried in a vacuum centrifuge and dissolved in 500 μl of the mobile phase (0.5 M phosphate buffer pH 7.0 and acetonitrile (85%:15%). One hundred μl of this solution was injected into the HPLC system (Tjaden *et al.*, 1987). Blood was centrifuged for 15 min at 2,000 r.p.m. and plasma was stored at − 30°C. Before analysis, the plasma was thawed and centrifuged at 6,000 r.p.m. The supernatant was diluted with water and 100 μl was injected into the HPLC system (Tjaden *et al.*, 1987).

*Toxicity study*

First the maximally tolerated dose for HAI was determined by assigning non-tumour-bearing rats to six groups: (1) sham HAI ($n$ = 4); (2) to (5) 1.2 ($n$ = 4), 1.5 ($n$ = 6), 2.4 ($n$ = 4), 3.6 ($n$ = 4) and 4.8 ($n$ = 4) mg MMC $kg^{-1}$ total body weight, respectively. Subsequently non-tumour-bearing rats were assigned to five groups for the ILP toxicity study: (1) sham ILP ($n$ = 2); (2) to (5) 1 ($n$ = 2), 3 ($n$ = 4), 4 ($n$ = 6), and 5 ($n$ = 4) times the maximally tolerated dose (1.2 mg $kg^{-1}$) for HAI, respectively.

*Tissue and plasma concentration study*

Tumour-bearing rats were randomly assigned to three treatment groups: (1) 1.2 mg $kg^{-1}$ ($n$ = 5) via bolus HAI; (2) 1.2 mg $kg^{-1}$ ($n$ = 6) and (3) 4.8 mg $kg^{-1}$ ($n$ = 6) as a bolus in the ILP circuit. During 25 min (perfusion (20 min) plus washout (5 min) time) liver and tumour tissue biopsies were taken. A liver biopsy was taken and a whole tumour was excised at 5, 15 and 20 min after administration of mitomycin C. From ILP as well as HAI treated rats a plasma sample was collected at $t$ = 25 min since at this time point the plasma concentration is maximal in ILP treated rats (de Brauw *et al.*, 1988). All samples were prepared for HPLC analysis.

To be able to calculate the tissue and plasma concentrations of mitomycin C in the samples that were collected during the study, samples for calibration lines were prepared identically and simultaneously: known concentrations of mitomycin C were added to liver, tumour and plasma derived from non-treated rats and immediately after the addition of mitomycin C the samples were prepared for HPLC analysis.

*Statistics*

Data were computerised for statistical analysis. Initially multivariate analysis of variance with repeated measurements was used to compare weight and tissue concentration changes across time versus the different treatment groups. Because of significant interaction across these factors and thus complex interpretation and description of the results, one-way analysis of variance at each time point is used in the present presentation to compare the means of these factors in the different groups. If significant differences were detected, a multiple range test, according to Scheffe, was performed. $P < 0.05$ was considered significant. To study the effect of the treatment on white blood cell count within each group, a paired $t$ test was used for each group to compare the white blood cell count at each time point with the starting value. For this procedure $P < 0.01$ was considered significant. Comparisons between the plasma concentrations were carried out using analysis of variance.

### Results

*Time–weight change curves*

*Hepatic artery infusion* The average changes in weight after mitomycin C treatment via HAI are illustrated in Figure 1a. Control HAI with saline 0.9% had no effect on body weight. Bolus HAI of 1.2 mg $kg^{-1}$ resulted in a weight dip (mean value 42 g) between days 8 and 12 and at about day 28 the rats had regained their starting weight. None of the 1.2 mg $kg^{-1}$ treated rats died. When intermediate doses (1.5 and 2.4 mg $kg^{-1}$) were administered the time–weight change curves were characterised by a triphasic pattern: a rapid weight loss, a steady state and a second fall in weight phase (Figure 1a). Comparing the 1.5 and 2.4 mg $kg^{-1}$ treated groups, the weight loss during the first phase was about 25 g less in the 1.5 mg $kg^{-1}$ group (57 versus 83 g) and the steady state phase was about 8 days longer (18 versus 10 days); it resulted in significantly ($P < 0.05$) less weight loss from day 17 until death in the 1.5 mg $kg^{-1}$ treated group than in the 2.4 mg $kg^{-1}$ treated group. In the 3.6 mg $kg^{-1}$ treated group one rat showed the triphasic pattern but the other three died 10–14 days after treatment after continuous rapid weight loss. Administration of 4.8 mg mitomycin C $kg^{-1}$ by HAI was lethal to all rats within 5 days. These rats were losing 10 g body weight per day.

*Isolated liver perfusion* In contrast to the HAI groups, all rats treated by ILP survived a mitomycin C dose of 4.8 mg $kg^{-1}$ (4 × maximally tolerated dose for HAI). These rats treated with high dose mitomycin C showed a dip in their weight between days 9 and 12 (mean values: 58 g after 3.6 mg $kg^{-1}$; 67 after 4.8 mg $kg^{-1}$). In contrast to the weight loss in HAI treated rats, not all of the weight loss in ILP treated rats was due to mitomycin C toxicity since a sham ILP caused a mean weight loss of 27 g. Results are illustrated in Figure 1b.

*Systemic and hepatic toxicity after HAI or ILP*

*Systemic toxicity* During the follow-up, white blood cell count was determined twice in the first week and then once a week together with the electrolytic status (sodium, potassium, urea and creatinine levels in serum).

Three days after administration of mitomycin C by HAI all rats had a significantly decreased white blood cell count ($P < 0.01$) (Figure 2a). At day 7 the white blood cell count regained normal values. In contrast in all ILP treated rats white blood cell count was significantly increased at day 3 ($P < 0.05$) (Figure 2b). Differences during the rest of the follow-up were not significant.

**Figure 2** Average WBC count ( $\times 10^9\,l^{-1}$) after administration of bolus MMC **a**, by hepatic artery infusion: + 0 ($n$ = 4), * 1.2 ($n$ = 4), □ 1.5 ($n$ = 6), × 2.4 ($n$ = 4) and ◇ 3.6 ($n$ = 4) mg MMC $kg^{-1}$ or **b**, in isolated liver perfusion: * 1.2 ($n$ = 2), ◇ 3.6 ($n$ = 4), Δ 4.8 ($n$ = 6) mg MMC $kg^{-1}$.

**Figure 3** Average serum level of urea determined once a week until death or to a maximum of 8 weeks in rats treated with bolus MMC: □ 1.5 ($n$ = 6) or × 2.4 ($n$ = 4) mg $kg^{-1}$ via the hepatic artery, or with Δ 4.8 ($n$ = 6) mg $kg^{-1}$ in isolated liver perfusion setting. The horizontal lines are the 5% and 95% limits of the normal value range. s.d. (vertical bars) is only indicated if at least one rat had a urea level higher than the 95% limit.

*Tissue and plasma concentration measurements*

The toxicity study showed that the maximally tolerated doses of mitomycin C in ILP and via HAI were 4.8 mg $kg^{-1}$ and 1.2 mg $kg^{-1}$ respectively. The concentration of mitomycin C in plasma was significantly lower in both ILP groups (1.2 mg $kg^{-1}$ group, 22 ng $ml^{-1}$; 4.8 mg $kg^{-1}$ group, 212 ng $ml^{-1}$) than in the HAI group (1.2 mg $kg^{-1}$ group, 539 ng $ml^{-1}$). The liver tissue concentrations of mitomycin C were of the same level in the two 1.2 mg $kg^{-1}$ groups, but were significantly higher in the ILP group with 4.8 mg $kg^{-1}$. The peak concentration in this 4.8 mg $kg^{-1}$ in ILP group was 3–4 times higher than in the two other groups (ILP 4.8 mg $kg^{-1}$ group, 2147 ng $g^{-1}$; ILP and HAI 1.2 mg $kg^{-1}$ groups, 668 and 671 ng $g^{-1}$).

In eight out of nine cases the mean tissue concentrations were higher in the poorly vascularised tumour (histologically determined, central necrosis present in tumours growing above 3–5 mm) than in liver tissue (Figure 5). This was most pronounced in the ILP with 4.8 mg $kg^{-1}$ group. At $t$ = 5 min the difference was significant ($P < 0.05$). The peak concentration in the ILP with 4.8 mg $kg^{-1}$ group was three and five times higher than in the ILP and HAI with 1.2 mg $kg^{-1}$ groups respectively (ILP 4.8 mg $kg^{-1}$ group, 3366 ng $g^{-1}$; ILP and HAI 1.2 mg $kg^{-1}$ groups, 1328 and 724 ng $g^{-1}$).

**Figure 4** Average serum level of bilirubin determined once a week until death or to a maximum of 8 weeks following MMC treatment in the isolated liver perfusion setting: * 1.2 ($n$ = 2), ◇ 3.6 ($n$ = 4) and Δ 4.8 ($n$ = 6) mg MMC $kg^{-1}$, or via bolus hepatic artery infusion: □ 1.5 ($n$ = 6) mg MMC $kg^{-1}$. The horizontal lines are the 5% and 95% limits of the normal value range. s.d. (vertical bars) is only indicated if at least one rat had a bilirubin level higher than the 95% limit. The kinetics of hepatic dysfunction seemed similar for serum glutamic-oxaloacetic transaminase and serum glutamic-pyruvic transaminase.

### Discussion

Dose increase of mitomycin C is limited by systemic toxic side-effects: delayed myelosuppression, pulmonary, cardiac

**Figure 5** Mean liver (■) and tumour (□) tissue concentrations of mitomycin C in three treatment groups: (1) HAI with 1.2 mg $kg^{-1}$ ($n$ = 5); (2) ILP with 1.2 mg $kg^{-1}$ ($n$ = 6) and (3) ILP with 4.8 mg $kg^{-1}$ ($n$ = 6). In each group biopsies of liver tissue and a whole tumour were taken at 5, 15 and 20 min after bolus administration of the dose and tissue concentrations of MMC were measured using HPLC.

and renal toxicity (Crooke & Bradner, 1976; Gunstream *et al.*, 1983; Chang *et al.*, 1986; Verweij *et al.*, 1987; Cattell, 1985). In the present study we first evaluated whether a higher dose of mitomycin C could be administered in ILP setting than via HAI and whether dose increase was limited by liver or systemic toxicity. Subsequently, rats were treated with the respective maximally tolerated doses of mitomycin C in ILP and HAI setting and mitomycin C concentrations were measured in liver tissue and in plasma to be able to correlate the differences in toxicity pattern with differences in tissue and plasma concentrations. Furthermore, tumour tissue concentrations were measured to ascertain that the higher doses resulted in higher tumour tissue concentrations. We demonstrated that quite high doses of mitomycin C could be delivered to the liver. All rats treated with 4.8 mg kg$^{-1}$ by ILP survived the 'therapy', while bolus infusion of 1.5 mg kg$^{-1}$ by HAI was already 100% lethal.

After ILP treatment with 4.8 mg kg$^{-1}$, SGOT and SGPT were 3–4 times normal during the first two post-operative weeks indicating some liver cell damage. Since the serum potassium levels were within normal range during the blood sample analysis the increased SGPT levels in this study were not due to haemolysis but to liver cell damage. Bilirubin was even more elevated (7–10 times normal) but turned to normal values within 6 weeks. These results indicate that in ILP setting 4.8 mg kg$^{-1}$ (four times the maximally tolerated dose in hepatic artery infusion setting) can still be tolerated. Toxicity is transient and confined to the liver. Five times the maximally tolerated dose in HAI setting (6.0 mg kg$^{-1}$) was 100% lethal within 3 days. Severe liver toxicitity led to multiple infarction and massive hepatocellular necrosis. In a clinical study evaluating intensive mitomycin C therapy and autologous bone marrow transplantation liver toxicity had been observed too. This therapy resulted in veno-occlusive disease of the liver in six out of 29 patients (Lazarus *et al.*, 1982). In our rat study autopsy did not show any of the characteristic signs of veno-occlusive disease of the liver in any rat.

Three days after ILP all rats had a significant increase in the white blood cell count instead of the significant decrease in white blood cell count that was seen in all rats treated with HAI. These results clearly demonstrate that in the ILP setting liver toxicity rather than systemic toxicity is dose-limiting.

When rats were treated with 1.2 mg kg$^{-1}$ via HAI the only signs of toxicity were weight loss during the first 2 weeks after bolus administration of mitomycin C and a white blood cell count dip in the first week. When rats were treated with doses higher than 1.2 mg kg$^{-1}$ again the myelosuppressive effect of mitomycin C was seen in the first week after HAI only. However, 4 weeks after administration of the lethal dose the urea levels started to increase and the rats died within 10 days with 3–4 times normal urea levels. The rise in serum urea levels concurred with the onset of the second fall in weight (Figure 1a). Severe weight loss resulting in increased protein catabolism could be responsible for this high urea level.

Macroscopic inspection at autopsy revealed no signs of the toxic side effects of mitomycin C as described by others: pulmonary toxicity (Gunstream *et al.*, 1983), cardiac toxicity (Levillain & Cluzan, 1973; Ganz *et al.*, 1983) and renal toxicity (Cattell, 1985; Verweij, 1986). Although the actual cause of death is unknown, rats treated with doses of mitomycin C higher than the maximally tolerated dose most probably were killed by delayed systemic toxicity.

The difference in toxicity pattern seen in ILP and HAI treated rats corresponded well with the differences in the concentrations measured in plasma and in liver tissue. Apparently there was minimal leakage from the isolated circuit to the systemic circulation, during and after perfusion, since the concentration of mitomycin C in plasma was significantly lower in the two ILP groups than in the HAI group. The liver tissue concentration in the rats treated with 1.2 mg kg$^{-1}$ was almost identical in the ILP and HAI groups and in both groups no sign of liver toxicity was observed. In contrast the much higher liver tissue concentration in the 4.8 mg kg$^{-1}$ treated rats obviously was toxic to the liver.

In this study the ILP technique (Brauw *et al.*, 1988) was extended with a cannula in the gastroduodenal artery as a second infusion limb in the perfusion circuit. This hepatic arterial infusion limb is essential since established liver metastases receive most of their blood supply from the hepatic artery (Ackerman, 1974; Izumi *et al.*, 1986; Sigurdson *et al.*, 1987). Employing this ILP technique, with two inflow limbs in the perfusion circuit, the tumour tissue concentrations were at least as high as the liver tissue concentrations. This is remarkable since the tumour tissue is poorly vascularised compared with the liver tissue. In the ILP with 4.8 mg kg$^{-1}$ group the mean tissue concentrations in tumour were even much higher than in liver ($t$ = 5 min, $P < 0.05$). A possible explanation for the relatively high tumour tissue concentrations could be a more rapid metabolism of mitomycin C in liver than in tumour tissue. The antitumour effect of ILP with mitomycin C is currently under investigation. Preliminary results indicate that ILP with the highest dose is very successful. Complete remissions are observed after ILP, but not after HAI (manuscript is in preparation).

Schneider *et al.* (1989) demonstrated that mitomycin C is still a drug of interest for chemotherapeutic treatment of colorectal cancer. They conclude that intrahepatic mitomycin C has a definite salvage benefit in some patients with hepatic metastases from colorectal carcinoma previously treated with intrahepatic FUDR. We conclude that if in man, like in the rats in this study, hepatic metastases can be exposed to much higher doses of mitomycin C by ILP, this therapeutic option may be of clinical value.

This study was supported by grant IKW 88-07 from the Dutch Cancer Foundation.

### References

ACKERMAN, N.B. (1974). The blood supply of experimental liver metastases IV Changes in vascularity with increasing tumor growth. *Surgery*, **75**, 589.

ADSON, M.A. (1983). Hepatic metastases in perspective. *Am. J. Roentgenol.*, **140**, 695.

ADSON, M.A., VAN HEERDEN, J.A., ADSON, M.H., WAGNER, J.S. & ILSTRUP, D.M. (1984). Resection of hepatic metastases from colorectal cancer. *Arch. Surg.*, **119**, 647.

AIGNER, K.R., WALTHER, H., TONN, J.C. & 4 others (1982). Die isolierte Leberperfusion mit 5-Fluorouracil (5-FU) beim Menschen. *Chirurg*, **53**, 571.

AIGNER, K.R., WALTHER, H. & LINK, K.H. (1988). Isolated liver perfusion with MMC/5-FU – surgical technique, pharmacokinetics, clinical results. *Contr. Oncol.*, **29**, 229.

AUGUST, D.A., SUGARBAKER, P.H., OTTOW, R.T., GIANOLA, F.J. & SCHNEIDER, P.D. (1985). Hepatic resection of colorectal metastases. Influence of clinical factors and adjuvant intraperitoneal 5-fluorouracil via Tenckhoff catheter on survival. *Ann. Surg.*, **201**, 210.

BALCH, C.M., URIST, M.M., SOONG, S.J. & MCGREGOR, M. (1983). A prospective phase II clinical trial of continuous FUDR regional chemotherapy for colorectal metastases to the liver using a totally implantable drug infusion pump. *Ann. Surg.*, **198**, 567.

DE BRAUW, L.M., VAN DE VELDE, C.J.H., TJADEN, U.R. & 4 others (1988). *In vivo* isolated liver perfusion technique in a rat hepatic metastasis model: 5-fluorouracil concentrations in tumor tissue. *J. Surg. Res.*, **44**, 137.

CATTELL, V. (1985). Mitomycin-induced hemolytic uremic kidney. An experimental model in the rat. *Am. J. Pathol.*, **121**, 88.

CHANG, A.Y., KUEBLER, J.P., PANDYA, K.J., ISRAEL, R.H., MARSHALL, B.C. & TORMEY, D.C. (1986). Pulmonary toxicity induced by mitomycin C is highly responsive to glucocorticoids. *Cancer*, **57**, 2285.

CHEN, H.S.G. & GROSS, J.F. (1980). Intra-arterial infusion of anticancer drugs: theoretic aspects of drug delivery and review of responses. *Cancer Treat. Rep.*, **64**, 31.

COLLINS, J.M. (1984). Pharmacologic rationale for regional drug delivery. *J. Clin. Oncol.*, **2**, 498.

CROOKE, S.T. & BRADNER, W.T. (1976). Mitomycin C: a review. *Cancer Treat. Rev.*, **3**, 121.

DE BRUIJN, E.A., SLEE, P.H.Th.J., KUPPEN, P.J.K. & 7 others (1988). The importance of exposure time in regional chemotherapy: mitomycin C and fluoropyrimidines. *Contr. Oncol.*, **29**, 43.

DOLL, D.C., WEISS, R.B. & ISSELL, B.F. (1985). Mitomycin: ten years after approval for marketing. *J. Clin. Oncol.*, **3**, 276.

FOSTER, J.M. & LUNDY, J. (1981). Liver metastases. *Curr. Probl. Surg.*, **18**, 157.

FOSTER, J.H. & BERMAN, M.M. (1977). Solid liver tumors. *Major Prob. Clin. Surg.*, **22**, 1.

GANZ, A., GOLD, B.S., TANDRON, I. & LURIE, K. (1983). Angina pectoris after doxorubicin and mitomycin C therapy. *Cancer Treat. Rep.*, **67**, 98.

GUNSTREAM, S.R., SEIDENFELD, J.J., SOBONYA, R.E. & MCMAHON, L.J. (1983). Mitomycin-associated lung disease. *Cancer Treat. Rep.*, **67**, 301.

IWATSUKI, S., ESQUIVEL, S.O., GORDON, R.D. & STARZL, T.E. (1986). Liver resection for metastatic colorectal cancer. *Surgery*, **100**, 804.

IYER, V.N. & SZYBALSKI, W. (1964). Mitomycins and porfiromycins: chemical mechanism of activation and cross-linking of DNA. *Science*, **145**, 55.

IZUMI, B., TASHIRO, S. & MIYAUCHI, Y. (1986). Anticancer effects of local administration of mitomycin C via the hepatic artery or portal vein on implantation and growth of VX2 cancer injected into rabbit liver. *Cancer Res.*, **46**, 4167.

KERPEL-FRONIUS, S., VERWEIJ, J., STUURMAN, M., KANYÁR, B., LELIEVELD, P. & PINEDO, H.M. (1988). Pharmacokinetics and toxicity of mitomycin C in rodents, given alone, in combination, or after induction of microsomal drug metabolism. *Cancer Chemother. Pharmacol.*, **22**, 104.

KUPPEN, P.J.K., SCHUITEMAKER, H., VAN'T VEER, L.J., DE BRUIJN, E.A., VAN OOSTEROM, A.T. & SCHRIER, P.I. (1988). *cis*-Diammine-dichloroplatinum(II)-resistant sublines derived from two human ovarian tumor cell lines. *Cancer Res.*, **48**, 3355.

LAZARUS, H.M., GOTTFRIED, M.R., HERZIG, W.H. & 8 others (1982). Veno-occlusive disease of the liver after high-dose mitomycin C therapy and autologous bone marrow transplantation. *Cancer*, **49**, 1789.

LEVILLAIN, R. & CLUZAN, R. (1973). Cardiac toxicity of antimitotic drugs. Proc. 3rd Meeting Eur. Assoc. Cancer Res., p. 100.

MARQUET, R.L., WESTBROEK, D.L. & JEEKEL, J. (1984). Interferon treatment of a transplantable rat colon adenocarcinoma: importance of tumor site. *Int. J. Cancer*, **33**, 689.

MOERTEL, C.G. (1978). Chemotherapy of gastrointestinal cancer. *N. Engl. J. Med.*, **299**, 1049.

SCHNEIDER, A., KEMENY, N., CHAPMAN, D., NIEDZWIECKI, D. & ODERMAN, P. (1989). Intrahepatic mitomycin C as a salvage treatment for patients with hepatic metastases from colorectal carcinoma. *Cancer*, **64**, 2203.

SCHWEMMLE, K., LINK, K.H. & RIECK, B. (1987). Rationale and indications for perfusion in liver tumors: current data. *World J. Surg.*, **11**, 534.

SIGURDSON, E.R., RIDGE, J.A., KEMENY, N. & DALY, J.M. (1987). Tumor and liver drug uptake following hepatic artery and portal vein infusion. *J. Clin. Oncol.*, **5**, 1836.

SKIBBA, J.L., ALMAGRO, K.A., CONDON, R.E. & PETROFF, R.J.A. (1983). A technique for isolation perfusion of the canine liver with survival. *J. Surg. Res.*, **34**, 123.

SLEE, P.H.Th.J., DE BRUIJN, E.A., LEEFLANG, P., KUPPEN, P.J.K., VAN DEN BERG, L. & VAN OOSTEROM, A.T. (1987). Variations in exposure to mitomycin C in an in vitro colony-forming assay. *Br. J. Cancer*, **54**, 951.

SUGARBAKER, P.H., GUNDERSON, L.L. & WITTES, R.E. (1985). Colorectal cancer. In *Cancer Principles and Practice of Oncology*, 2nd edn, DeVita, V.T., Hellman, S. & Rosenberg, S.A. (eds) p. 795. J.B. Lippincott: Philadelphia.

TJADEN, U.R., DE BRUIJN, E.A., VAN DER HOEVEN, R.A.M., JOL, C., VAN DER GREEF, J. & LINGEMAN, H. (1987). Automated analysis of MMC in body fluids by high performance liquid chromatography with on line sample pretreatment. *J. Chromatogr.*, **420**, 53.

VAN DE VELDE, C.J.H., DE BRAUW, L.M., SUGARBAKER, P.H. & TRANBERG, K.-G. (1987). Hepatic arterial infusion chemotherapy: rationale, results, credits and debits. In *Progress in Surgery of the Liver, Pancreas and Biliary System*. Bengmark, S. (ed.) p. 163. Martinus Nijhoff: Dordrecht.

VAN DE VELDE, C.J.H., KOTHUIS, B.J.L., BARENBRUG, H.W.M. & 4 others (1986). A successful technique of in-vivo isolated liver perfusion in pigs. *J. Surg. Res.*, **41**, 593.

VERWEIJ, J., VAN DER BURG, M.E.L. & PINEDO, H.M. (1987). Mitomycin C-induced hemolytic uremic syndrome. Six case reports and review of the literature on renal, pulmonary and cardiac side effects of the drug. *Radiother. Oncol.*, **8**, 33.

VERWEIJ, J. (1986). Studies on the toxicology of mitomycin C. Thesis, Free University of Amsterdam, The Netherlands.

WALLNER, K.E. & LI, G.C. (1987). Effect of drug exposure duration and sequencing on hyperthermic potentiation of mitomycin C. *Cancer Res.*, **47**, 493.